# ENIGMA‐DTI: Translating reproducible white matter deficits into personalized vulnerability metrics in cross‐diagnostic psychiatric research

**DOI:** 10.1002/hbm.24998

**Published:** 2020-04-16

**Authors:** Peter Kochunov, L. Elliot Hong, Emily L. Dennis, Rajendra A. Morey, David F. Tate, Elisabeth A. Wilde, Mark Logue, Sinead Kelly, Gary Donohoe, Pauline Favre, Josselin Houenou, Christopher R. K. Ching, Laurena Holleran, Ole A. Andreassen, Laura S. van Velzen, Lianne Schmaal, Julio E. Villalón‐Reina, Carrie E. Bearden, Fabrizio Piras, Gianfranco Spalletta, Odile A. van den Heuvel, Dick J. Veltman, Dan J. Stein, Meghann C. Ryan, Yunlong Tan, Theo G. M. van Erp, Jessica A. Turner, Liz Haddad, Talia M. Nir, David C. Glahn, Paul M. Thompson, Neda Jahanshad

**Affiliations:** ^1^ Maryland Psychiatric Research Center, Department of Psychiatry University of Maryland School of Medicine Baltimore Maryland USA; ^2^ Psychiatry Neuroimaging Laboratory, Brigham & Women's Hospital Boston Massachusetts USA; ^3^ Imaging Genetics Center USC Mark and Mary Stevens Neuroimaging & Informatics Institute, Keck School of Medicine of USC Marina del Rey California USA; ^4^ Department of Neurology University of Utah School of Medicine Salt Lake City Utah USA; ^5^ George E. Wahlen VA Salt Lake City Utah USA; ^6^ Brain Imaging and Analysis Center, Duke University Durham North Carolina USA; ^7^ VA Boston Healthcare System, National Center for PTSD Boston Massachusetts USA; ^8^ Boston University School of Medicine Department of Psychiatry Boston Massachusetts USA; ^9^ Boston University School of Medicine, Biomedical Genetics Boston Massachusetts USA; ^10^ Boston University School of Public Health Department of Biostatistics Boston Massachusetts USA; ^11^ Harvard Medical School Boston Massachusetts USA; ^12^ Centre for Neuroimaging and Cognitive Genomics (NICOG), Clinical Neuroimaging Laboratory NCBES Galway Neuroscience Centre, National University of Ireland Galway Galway Ireland; ^13^ Neurospin, CEA, Université Paris‐Saclay Gif‐sur‐Yvette France; ^14^ INSERM Unit U955, team “Translational Neuro‐Psychiatry”, Créteil France; ^15^ Psychiatry Department Assistance Publique‐Hôpitaux de Paris (AP‐HP), CHU Mondor Créteil France; ^16^ Faculté de Médecine, Université Paris Est Créteil Créteil France; ^17^ Norwegian Centre for Mental Disorders Research (NORMENT), Division of Mental Health and Addiction Oslo University Hospital Oslo Norway; ^18^ Norwegian Centre for Mental Disorders Research (NORMENT) Institute of Clinical Medicine, University of Oslo Oslo Norway; ^19^ Centre for Youth Mental Health, The University of Melbourne Melbourne Australia; ^20^ Orygen, The National Centre of Excellence in Youth Mental Health Parkville Australia; ^21^ Department of Psychiatry and Biobehavioral Sciences Semel Institute for Neuroscience and Human Behavior, University of California at Los Angeles Los Angeles California USA; ^22^ Department of Psychology University of California at Los Angeles Los Angeles California USA; ^23^ Laboratory of Neuropsychiatry, Department of Clinical and Behavioral Neurology IRCCS Santa Lucia Foundation Rome Italy; ^24^ Division of Neuropsychiatry, Menninger Department of Psychiatry and Behavioral Sciences Baylor College of Medicine Houston Texas USA; ^25^ Amsterdam UMC, Vrije Universiteit Amsterdam, Department of Psychiatry, Department of Anatomy & Neurosciences Amsterdam Neuroscience Amsterdam The Netherlands; ^26^ Department of Psychiatry & Neuroscience Institute, University of Cape Town SA MRC Unit on Risk & Resilience in Mental Disorders Cape Town South Africa; ^27^ Beijing Huilongguan Hospital, Peking University Huilongguan Clinical Medical School Beijing China; ^28^ Clinical Translational Neuroscience Laboratory, Department of Psychiatry University of California Irvine Irvine California USA; ^29^ Center for the Neurobiology of Learning and Memory University of California Irvine Irvine California USA; ^30^ Department of Psychology and Neuroscience Institute Georgia State University Atlanta Georgia USA; ^31^ Department of Psychiatry Boston Children's Hospital and Harvard Medical School Boston Massachusetts USA; ^32^ Olin Neuropsychiatric Research Center, Hartford Hospital Hartford Connecticut USA

**Keywords:** big data, cross‐disorder, DTI, ENIGMA, RVI, white matter deficit patterns

## Abstract

The ENIGMA‐DTI (diffusion tensor imaging) workgroup supports analyses that examine the effects of psychiatric, neurological, and developmental disorders on the white matter pathways of the human brain, as well as the effects of normal variation and its genetic associations. The seven ENIGMA disorder‐oriented working groups used the ENIGMA‐DTI workflow to derive patterns of deficits using coherent and coordinated analyses that model the disease effects across cohorts worldwide. This yielded the largest studies detailing patterns of white matter deficits in schizophrenia spectrum disorder (SSD), bipolar disorder (BD), major depressive disorder (MDD), obsessive–compulsive disorder (OCD), posttraumatic stress disorder (PTSD), traumatic brain injury (TBI), and 22q11 deletion syndrome. These deficit patterns are informative of the underlying neurobiology and reproducible in independent cohorts. We reviewed these findings, demonstrated their reproducibility in independent cohorts, and compared the deficit patterns across illnesses. We discussed translating ENIGMA‐defined deficit patterns on the level of individual subjects using a metric called the regional vulnerability index (RVI), a correlation of an individual's brain metrics with the expected pattern for a disorder. We discussed the similarity in white matter deficit patterns among SSD, BD, MDD, and OCD and provided a rationale for using this index in cross‐diagnostic neuropsychiatric research. We also discussed the difference in deficit patterns between idiopathic schizophrenia and 22q11 deletion syndrome, which is used as a developmental and genetic model of schizophrenia. Together, these findings highlight the importance of collaborative large‐scale research to provide robust and reproducible effects that offer insights into individual vulnerability and cross‐diagnosis features.

## 1 INTRODUCTION

The Enhancing Neuro Imaging Genetics through Meta‐Analysis (ENIGMA) Consortium was conceived in 2009 with the goal of performing large‐scale neuroimaging genetic studies and has since grown into a collaboration of more than 1,400 scientists worldwide (Thompson et al., 2013). The ENIGMA diffusion imaging working group was organized in 2009 to develop analytic workflows that analyze the effects of genes, environment, and neuropsychiatric disorders on white matter microarchitecture. The initial focus was on the multisite analysis of fractional anisotropy (FA) images, as this is the most commonly studied scalar parameter extracted from diffusion tensor imaging (DTI) (Basser, Mattiello, & LeBihan, 1994; Pierpaoli & Basser, 1996). The absolute FA values are sensitive to fiber coherence and organization, myelination levels, and axonal integrity and have been widely used as an index of white matter health (Thomason & Thompson, 2011). FA has emerged as a sensitive index of normal white matter maturation and aging (Penke, Munoz Maniega, Houlihan, et al., 2010; Penke, Munoz Maniega, Murray, et al., 2010). Prior to the ENIGMA studies, microstructural abnormalities were reported in many neuropsychiatric illnesses and brain disorders including schizophrenia spectrum disorder (SSD) (Alba‐Ferrara & de Erausquin, 2013; Friedman et al., 2008; Mandl et al., 2013; Nazeri et al., 2013), bipolar disorder (BD) (Barysheva, Jahanshad, Foland‐Ross, Altshuler, & Thompson, 2013; Sprooten et al., 2011), major depressive disorder (MDD) (Carballedo et al., 2012) and others. To date, the ENIGMA‐DTI protocols have been used in the largest studies ranking effect sizes for case–control differences in six common neuropsychiatric disorders and a genetic microdeletion syndrome (Table 1). We review the workflow used to derive these findings, and how their high reproducibility provides a basis for individual‐level measurements of microstructural signatures, thereby enabling neuropsychiatric research across diagnostic boundaries (Jahanshad et al., 2013).

**TABLE 1:** The number of subjects and cohorts that were used to derive disorder specific patterns for patient control differences

Disorder	*N*‐subjects (patients/controls)	*N*‐cohorts	Citation
SSD	*N* = 4,322 (1963/2359)	29	(Kelly et al., 2018)
BD	*N* = 3,033 (1,482/1551)	26	(Favre et al., 2019)
MDD	*N* = 2,907 (1,305/1602)	20	(van Velzen et al., 2019)
22q11DS	*N* = 594 (334 /260)	10	(Villalón‐Reina et al., 2019)
PTSD	*N* = 3,049 (1,446/1,603)	28	(Dennis et al., 2019)
OCD	*N* = 1,345 (700/645)	19	(Piras et al., 2019)
TBI	*N* = 705 (437/268)	5	(Dennis et al., 2018)

Abbreviations: BD, bipolar disorder; ENIGMA, Enhancing Neuro Imaging Genetics through Meta‐Analysis; MDD, major depressive disorder; OCD, obsessive–compulsive disorder; SSD, schizophrenia spectrum disorder; PTSD, posttraumatic stress disorder; TBI, traumatic brain injury.

## 2 ENIGMA‐DTI WORKFLOW

The ENIGMA‐DTI workflow provided a generalizable analysis approach to extract phenotypes from DTI data collected by imaging groups around the world (Jahanshad et al., 2013). This workflow is based on tract‐based spatial statistics (TBSS) (Smith et al., 2006), that uses a skeleton of major white matter tracts as the basis for determining statistical differences in regional FA values. The ENIGMA‐DTI protocol adapts the TBSS approach for performing ROI‐based multisite research by providing a custom protocol that includes QA/QC steps, a custom ENIGMA‐DTI minimal deformation warping target along with the skeleton of major white matter tracts, and steps to extract tract‐average FA values (Jahanshad et al., 2013). Diffusion measures extracted using the ENIGMA‐DTI workflow showed excellent reproducibility in both test–retest (McGuire et al., 2017) and longitudinal data (Acheson et al., 2017).

The inaugural aim of the workflow was to perform multisite heritability analyses of these quantitative DTI‐based phenotypes. We demonstrated that tractwise diffusion measures extracted using this workflow were consistently heritable (*h* ^2^ = 0.42–0.75)—regardless of the data collection protocol and study designs that included twins and siblings, extended families and pedigree‐based cohorts (Jahanshad et al., 2013; Kochunov, Fu, et al., 2016). The regional heritability patterns in data collected using different DTI protocols were likewise strongly correlated with each other (*r* ~ 0.6–0.9) (Kochunov, Fu, et al., 2016; Kochunov et al., 2015; Kochunov, Jahanshad, et al., 2014; Kochunov, Patel, et al., 2019). The high reproducibility and consistent heritability of ENIGMA‐DTI measures across diverse study designs and data collection protocols provided a strong rationale for disorder‐oriented ENIGMA working groups to use this workflow to map deficit patterns in studies of several major neuropsychiatric illnesses (Table 1).

## 3 ENIGMA‐DTI FINDINGS IN NEUROPSYCHIATRIC DISORDERS

To date, the ENIGMA‐DTI workflow was used to elucidate regional patient‐control differences in brain microstructure in SSD (Kelly et al., 2018), MDD (van Velzen et al., 2019), BD (Favre et al., 2019), obsessive compulsive disorder (OCD) (Piras et al., 2019), traumatic brain injury (TBI) (Dennis et al., 2018), posttraumatic stress disorder (PTSD) (Dennis et al., 2019), and 22q11 deletion syndrome (Villalón‐Reina et al., 2019) (Table 1). The results are reported as Cohen's *d*‐value effect sizes for the average FA values and for 24 regional tract‐wise measurements. The average FA was calculated for the entire white matter skeleton and their effect sizes are informative of the whole‐brain effect sizes. The average FA values include the values for regional measurements that constitute about 20% of the total skeletons. The effect sizes were derived using the largest samples of the respective disorders available to date and consisted of hundreds to thousands of patients and controls. The use of the ENIGMA‐DTI workflow across sites and disorders led to harmonized analyses of data and the reporting of effect sizes (Table 2). The triad of the most debilitating major psychiatric illnesses: SSD, BD, and MDD were characterized by highly significant reductions in the average FA values in patients compared with controls (Table 2). The largest effect size for the average FA values was observed for SSD (Cohen's *d* = −0.42, *p* = 4·10^−24^). The patients with BD and MDD showed significant and similar negative effect sizes on the average FA values (Cohen's *d* = −0.26, *p* ≤ 10^−3^) (Table 2). Patients with SSD, BD, and MDD also showed a pattern of significant regional reductions in FA values. The comparison of regional effect sizes across the disorders provided a unique opportunity to summarize the impact of these illnesses across diagnostic categories (discussed in Section 4.2). Other illnesses did not show significant patient–control differences for the average FA values. Patients with OCD showed a modest number of regions, including the sagittal stratum (SS) and posterior thalamic radiation (PTR), where cases on average, had lower FA than controls (Table 2). Subjects with PTSD showed no difference in either average (Cohen's *d* = −0.02, *p* = .7) or regional FA values (Table 2). Subjects with the 22q11 deletion syndrome showed no significant difference in average FA values from controls (Cohen's *d* = 0.09, *p* = .3), yet there were large regional effect sizes in both directions. On average, cases had *higher* FA values compared to controls in the callosal and cortico‐thalamic tracts and *lower FA* in the fornix‐stria terminalis (FX‐ST), superior longitudinal fasciculus (SLF), and the external/extreme capsules (EC). 22q11 deletion syndrome is a chromosomal microdeletion syndrome that greatly elevates risk for psychosis and schizophrenia. A finding of higher FA values in frontal areas in 22q11 deletion subjects stands in contrast with generally negative effects on FA observed in the three major neuropsychiatric disorders. However, it is consistent with postmortem histological examinations that showed higher cumulative cellular membrane circumference of cerebral white matter axons in 22q11 deletion patients (Villalón‐Reina et al., 2019). Subjects with TBI (primarily mild TBI) likewise showed nonsignificant effects in the average FA (Cohen's *d* = 0.12, *p* = .20) and chiefly positive but nonsignificant regional effect sizes (higher FA values) when compared with controls (Table 2). This finding was interpreted as a possible marker of recovery by the original study (Dennis et al., 2018). Together, these findings provide the first opportunity to evaluate the cross‐disorder similarity, especially if these findings are reproducible in the independent samples.

**TABLE 2:** Meta‐analytical effect sizes (Cohen's *d*‐values) (with group‐wise significance in parentheses) of the patients versus control differences in disorders studied by ENIGMA disorder‐oriented workgroups. The sample information for each disorder is provided in Table 1

Region	SSD	BD	MDD	22q11DS	PTSD	OCD	TBI
Average FA	−0.42 (4·10^−24^)	−0.26 (6·10^−4^)	−0.26 (1·10^−3^)	0.09 (0.3)	−0.02 (0.7)	−0.20 (0.07)	0.12 (0.2)
Anterior corona radiata (ACR)	−0.40 (9·10^19^)	−0.24 (1·10^−6^)	−0.25 (1·10^−3^)	0.23 (0.1)	−0.01 (0.9)	−0.12 (0.2)	0.04 (0.7)
Anterior limb of internal capsule (ALIC)	−0.37 (2·10^−15^)	−0.15 (4·10^−3^)	−0.23 (4·10^−3^)	0.64 (1·10^−12^)	−0.04 (0.3)	−0.06 (0.6)	0.04 (0.7)
Body of corpus callosum (BCC)	−0.39 (2·10^−18^)	−0.43 (2·10–16)	−0.24 (2·10^−3^)	0.37 (3·10^−5^)	−0.04 (0.30)	−0.11 (0.30)	0.03 (0.7)
Corpus callosum (CC)	−0.40 (8·10^−19^)	−0.46 (5·10^−17^)	−0.25 (2·10^−3^)	0.54 (1·10^−9^)	−0.05 (0.2)	−0.16 (0.1)	0.002 (0.9)
Cingulum (cingulate gyrus part) CGC	−0.27 (3·10^−9^)	−0.39 (6·10^−11^)	−0.17 (2·10^−3^)	0.20 (0.04)	−0.03 (0.4)	−0.06 (0.4)	−0.01 (0.9)
Perihippocampal cingulum tract (CGH)	−0.11 (0.01)	−0.07 (0.14)	−0.07 (0.14)	−0.45 (1·10^−4^)	0.02 (0.7)	−0.07 (0.3)	0.07 (0.4)
Corona radiata (CR)	−0.33 (3·10^−17^)	−0.20 (4·10^−4^)	−0.25 (2·10^−3^)	0.38 (1·10^−5^)	−0.02 (0.5)	−0.13 (0.3)	0.09 (0.3)
Cortico‐spinal tract (CST)	−0.04 (0.24)	0.00 (1.00)	−0.10 (0.10)	0.05 (0.7)	0.03 (0.5)	0.06 (0.3)	−0.14 (0.2)
External capsule (EC)	−0.21 (1·10^−7^)	−0.23 (4·10^−7^)	−0.16 (0.02)	−0.47 (1·10^−4^)	0.03 (0.4)	−0.12 (0.2)	0.20 (0.02)
Fornix (FX)	−0.31 (7·10^−12^)	−0.29 (8·10^−8^)	−0.08 (0.09)	−0.74 (1·10^−13^)	−0.02 (0.7)	−0.11 (0.2)	0.08 (0.4)
Fornix/Stria terminalis (FXST)	−0.32 (8·10^−14^)	−0.16 (7·10^−5^)	−0.18 (3·10^−3^)	−0.30 (0.01)	0.00 (1.0)	−0.08 (0.4)	−0.04 (0.7)
Genu of corpus callosum (GCC)	−0.37 (1·10^−18^)	−0.37 (2·10^−8^)	−0.25 (1·10^−3^)	0.58 (4·10^−9^)	−0.01 (0.8)	−0.17 (0.04)	−0.01 (0.9)
Internal capsule (IC)	−0.18 (2·10^−5^)	−0.07 (0.2)	−0.23 (0.01)	0.68 (1·10^−13^)	0.00 (0.9)	−0.02 (0.9)	0.03 (0.7)
Uncinate fasciculus (UNC)	−0.11 (0.004)	−0.19 (2·10^−6^)	−0.12 (0.01)	0.03 (0.7)	0.02 (0.6)	−0.04 (0.6)	0.12 (0.2)
Posterior corona radiata (PCR)	−0.25 (2·10^−12^)	−0.15 (3·10^−3^)	−0.20 (4·10^−3^)	0.52 (6·10^−9^)	−0.04 (0.3)	−0.16 (0.02)	0.18 (0.03)
Posterior limb of internal capsule (PLIC)	0.04 (0.37)	0.04 (0.5)	−0.15 (0.08)	0.81 (2·10^−15^)	0.03 (0.5)	0.04 (0.6)	0.01 (0.9)
Posterior thalamic radiation (PTR)	−0.31 (1·10^−18^)	−0.30 (3·10^−12^)	−0.14 (0.12)	−0.01 (0.9)	−0.03 (0.5)	−0.26 (1·10^−3^)	0.12 (0.3)
Retrolenticular limb of the internal capsule (RLIC)	−0.13 (0.002)	−0.05 (0.40)	−0.15 (0.05)	0.20 (0.07)	0.00 (1.00)	−0.03 (0.8)	0.04 (0.6)
Splenium of corpus callosum (SCC)	−0.22 (4·10^−6^)	−0.34 (2·10^−10^)	−0.13 (0.04)	0.44 (2·10^−4^)	−0.08 (0.10)	−0.12 (0.2)	0.02 (0.9)
Superior corona radiata (SCR)	−0.15 (7·10^−6^)	−0.09 (0.13)	−0.20 (0.02)	0.26 (3·10^−3^)	−0.02 (0.6)	−0.07 (0.4)	0.12 (0.2)
Superior fronto‐occipital fasciculus (SFO)	−0.29 (4·10^−8^)	−0.15 (4·10^−3^)	−0.23 (4·10^−3^)	−0.12 (0.5)	−0.10 (0.01)	−0.08 (0.3)	0.06 (0.50)
Superior longitudinal fasciculus (SLF)	−0.22 (6·10^−8^)	−0.23 (5·10^−6^)	−0.17 (0.04)	−0.32 (2·10^−4^)	0.03 (0.5)	−0.12 (0.3)	0.26 (0.003)
Sagittal stratum (SS)	−0.30 (5·10^−14^)	−0.20 (8·10^−5^)	−0.23 (4·10^−3^)	0.08 (0.5)	−0.02 (0.6)	−0.21 (0.001)	0.09 (0.4)
Tapetum (TAP)	−0.16 (9·10^−7^)	−0.25 (2·10^−6^)	−0.12 (0.17)	0.86 (6·10^−21^)	−0.11 (0.01)	−0.18 (0.01)	0.14 (0.09)

Abbreviations: BD, bipolar disorder; ENIGMA, Enhancing Neuro Imaging Genetics through Meta‐Analysis; FA, fractional anisotropy; MDD, major depressive disorder; OCD, obsessive–compulsive disorder; SSD, schizophrenia spectrum disorder; PTSD, posttraumatic stress disorder; TBI, traumatic brain injury.

## 4 REPRODUCIBILITY OF ENIGMA FINDINGS IN NEUROPSYCHIATRIC DISORDERS

Research findings in neuropsychiatric illnesses have historically suffered from a substantial variability and heterogeneity both within and across disorders including genetics, environmental risk factors, mean age of onset, symptom presentations, treatment response, and long‐term prognosis. The sources of heterogeneity have long remained elusive to clinicians and scientists and have contributed to a surprisingly poor reproducibility of neuroanatomical, functional, and genetic findings in neuropsychiatric illnesses. Meta‐analysis has always offered a principled approach to screen studies for false positive findings by overcoming the “chasing of significance” observed in some discovery studies (Ioannidis, 2014). The big data analyses performed by ENIGMA differ from the traditional meta‐analytic studies that derive the mean effect from group‐level comparisons based on *previously published* effect sizes and often fall prey to the heterogeneity of the underlying methods used in the original studies. Instead, ENIGMA analyses are more akin to the “multisite‐study analytic” approaches that directly coordinate the analysis of many data sets, by a group of collaborating scientists using the methods vetted for multisite research. However, ENIGMA does not enforce an a priori selection of image acquisition protocols and behavioral or diagnostic assessments. Instead, ENIGMA pays considerable attention at each participating site to ensure the quality, integrity, and homogeneity of the underlying data, validity of the outcomes, and reproducibility of the deficit patterns.

A study by the ENIGMA‐schizophrenia workgroup on subcortical deficits was the first validation of large‐scale cooperative analyses of neuroimaging data in a severe mental illness. It used standardized methods to assess a sample of 2,028 patients and 2,540 controls from 15 centers worldwide (van Erp et al., 2015). This was the first study to show that the effect size for the smaller hippocampus in SSD patients was greater than that for the well‐known enlargement of the lateral ventricles, refocusing attention on the neurological basis of this disorder. It also provided the first opportunity to test the premise that Big Data neuroimaging approaches could improve the reproducibility of findings in a disorder known for its heterogeneity. In a recent editorial, we observed that the effect sizes for patient–control group differences for volumes of subcortical structures reported by the ENIGMA‐schizophrenia group were in remarkable correlation (*r* ^2^ > 0.9) with two studies performed since then in largely independent cohorts (Alnaes et al., 2019; Kochunov, Thompson, & Hong, 2019; Okada et al., 2016).

The ENIGMA‐schizophrenia group followed up with the study of white matter alterations based on a sample of 1,963 patients and 2,359 healthy controls from 29 independent international cohorts (Kelly et al., 2018, Holleran et al., 2020). This study was the first to report a regional localization of deficits in this illness as the regional pattern of effect sizes. The associative white matter tracts that connect the frontal, parietal, temporal, and limbic areas such as the anterior *corona radiata* (ACR) and the body and *genu* of the corpus callosum (GCC), showed significantly lower FA values in individuals with schizophrenia compared with controls. In contrast, cerebral pathways that carry sensorimotor fibers, such as the corticospinal tract and posterior limb of the internal capsule, showed no detectable patient–control group differences (Table 2). Importantly, patients diagnosed with schizophrenia also had significantly lower integrity of the fornix (FX)—the primary tract connecting the hippocampus with the frontal brain regions, consistent with the anatomical specificity observed for the subcortical volumetric deficits.

**FIGURE 1:**
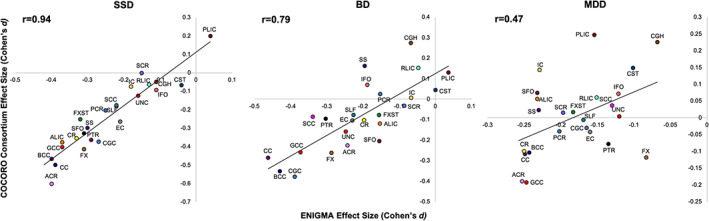
Scatter plot of regional effect sizes (Cohen's d coefficients) calculated for SSD (left), BD (center) and MDD (right) by COCORO consortium (*y*‐axis) versus ENIGMA workgroup reports (*x*‐axis). The effect sizes calculated in nonoverlapping cohorts showed very strong correlation for SSD (*r* = 0.94), strong correlation for BD (*r* = 0.79) and moderate correlation for MDD (*r* = 0.47). BD, bipolar disorder; ENIGMA, Enhancing Neuro Imaging Genetics through Meta‐Analysis; MDD, major depressive disorder; SSD, schizophrenia spectrum disorder

The regional pattern of white matter deficits reported by the ENIGMA illness‐focused working groups has since been replicated by several independent studies, including a recent meta‐analysis study from the Japanese Cognitive Genetics Collaborative Research Organization (COCORO) consortium. The Social Processes Initiative in the Neurobiology of Schizophrenia (SPINS) study showed that regression of site‐specific sources of methodological variance—such as day‐to‐day variations in scanner magnetic field gradients, RF coils, and electronics performance measured using a diffusion phantom—significantly improved the agreement between white matter deficit patterns in the SPINS sample and the ENIGMA‐schizophrenia pattern; correlations increased from 0.55 to 0.81 (Kochunov, Dickie Erin, et al., 2018). Kochunov and colleagues showed that the ENIGMA‐schizophrenia pattern was very highly correlated (*r* = 0.92) with the measured deficit pattern in another cohort and partly explained the two chief cognitive deficits in SSD: processing speed and working memory (Kochunov et al., 2017). A report on findings from the Beijing Connectome Project (BCP) found a high correlation (*r* = 0.86) between regional effect sizes observed in a sample of Han Chinese and the ENIGMA‐schizophrenia pattern. A COCORO study used the ENIGMA‐DTI workflow and an independent cohort collected across 12 sites in Japan to calculate regional effect sizes for SSD (*N* = 696 patients), BD (*N* = 211 patients), and MDD (*N* = 398 patients) using *N* = 1,506 healthy controls (Koshiyama et al., 2019). We report a very high correlation in regional effect sizes by ENIGMA and COCORO for SSD (*r* = 0.94), high correlation for effect sizes of BD (*r* = 0.79) and moderate correlation for MDD (*r* = 0.47) (Figure 1). The magnitudes of regional effect sizes reported by ENIGMA and COCORO showed no significant differences (paired *t*‐test) for SSD (*p* = .9). ENIGMA regional effect sizes were significantly higher for both BD (average Cohen's *d* = −0.21 ± 0.03 vs. −0.08 ± 0.03, for ENIGMA and COCORO, respectively, *p* = 2·10^−6^) and MDD (average Cohen's *d* = −0.18 ± 0.01 vs. 0.00 ± 0.02, for ENIGMA and COCORO, respectively, *p* = 2·10^−9^). In summary, big data neuroimaging studies can derive patterns of neuroanatomical deficits in neuropsychiatric illnesses. The deficit patterns for SSD and BD were significant and showed excellent‐to‐good consistency and reproducibility across geographically and ethnically diverse cohorts. The deficit patterns for MDD showed a moderate consistency, likely due to more modest effect sizes; however, this may improve once more independent studies are conducted.

### 4.1 Translating ENIGMA findings to the individual level

The excellent agreement observed between the ENIGMA regional deficit patterns provides a novel perspective on big data neuroimaging findings. The inclusive worldwide nature of these studies has likely removed site‐specific variances in diagnosis, medication, and environment, yielding deficit patterns that remain even after treatment with existing therapies and are shared by patients worldwide. The remarkable agreement across cohorts within each SSD meta‐analysis, and their subsequent independent replication studies, indicates that the profile of these regional effect sizes may be a signature, or a vector, that is related to the signature of the common physiopathological processes in schizophrenia or currently unmet treatment targets including cognitive deficits, treatment resistance, symptoms, and others. We first utilized the ENIGMA‐schizophrenia DTI pattern as a predictor in the structural equation modeling of two major cognitive deficits, processing speed and working memory, that are affected in SSD patients. We found that the individual similarity with the ENIGMA‐schizophrenia deficit pattern mediated the association between white matter abnormalities in individual patients and the severity of cognitive deficits (Kochunov et al., 2017). The same pattern of structure–function association was also observed in controls. This suggested that the regional pattern of the schizophrenia‐related white matter deficits predicted the association between white matter and cognition even in the controls, indicating that the cognitive effects in schizophrenia are likely driven by reduced white matter integrity that are not secondary effects of antipsychotic medications (Kochunov et al., 2017).

The next step is to translate the findings from ENIGMA studies to enable predictions of vulnerability at the individual level. Can we use the characteristic patterns of regional deficits as predictors to link individual brain scans to vulnerability for a disorder and to its genetic risks, cognitive deficits and clinical features? Population genetic studies have developed polygenic risk scores (PRS) (Choi, Heng Mak, & O'Reilly, 2018), and SSD, BD, MDD, and OCD are highly heritable polygenic disorders with a complex pattern of gene by environment risk interactions. The genetic risks are conferred by many alleles detectable by genome‐wide association studies. PRS is calculated as a weighted linear combination of the alleles determined to either confer risk or act as protective factors for the illness, where the weights are assigned based on allele effect sizes and population frequencies (Choi et al., 2018). PRS was shown to be a better predictor of risk than any single candidate risk allele (Colodro‐Conde et al., 2018).

The regional vulnerability index (RVI) was developed as a simple correlational approach to quantify the agreement between an individual's brain and the expected pattern for the disorder. In contrast to PRS, the RVI approach is based on effect sizes derived from ethnically diverse samples and therefore RVI values are translatable across ethnicities (Kochunov, Huang, et al., 2019). RVI is a correlation coefficient between the normalized regional measures in an individual, such as tractwise FA or cortical gray matter thickness values, and the pattern of regional effect sizes reported by ENIGMA. A normalization process is used before computing the index, which includes a linear regression to remove effects of covariates, such as age and sex, from the individual's data, followed by *z*‐transforming the residuals using the average and *SD* calculated from the healthy controls. For each subject, this produces a vector of regional measurements that captures the deviation from the normative values for each brain region and therefore mimics the contrast captured by the Cohen's *d*‐values reported by ENIGMA. Higher RVI values (with a maximum of 1.0) indicate a better correlation with the expected disorder pattern. We hypothesized that higher similarity to the expected pattern is indicative of individual vulnerability to a disorder (Kochunov, Huang, et al., 2019).

We evaluated the RVI calculated for white matter DTI as a marker of treatment resistance in SSD (Kochunov, Huang, et al., 2019). The link between treatment resistance and cerebral white matter in SSD was suggested by previous white matter volume reduction findings (Molina et al., 2005) and reduced FA values (Holleran et al., 2014; Vanes, Mouchlianitis, Wood, & Shergill, 2018). In our study, we observed that RVI in treatment resistant patients was significantly higher than in patients who responded to treatment. Yet, no individual white matter region could consistently separate the treatment‐resistant and treatment‐responsive patients. This suggested that ENIGMA‐schizophrenia pattern in white matter may capture the deficits in this illness that do not improve with treatment. In Kochunov and colleagues' manuscript (published in this issue, Kochunov et al., 2020) we present the findings of white matter, cortical, and subcortical RVI in SSD. We show that the “agreement” with the respective SSD patterns can be used as a novel biomarker that is independent of the absolute differences in regional traits. Domain‐specific RVI values were significantly correlated with cognition and negative symptoms, even in the absence of significant correlation in the individual traits from that neuroimaging domain. It is not immediately clear why RVI captures individual variance in cognitive deficits and symptoms severity, but individual regional measures do not. Higher RVI‐SSD values likely reflect the contrast between the high vulnerability of associative and the lower vulnerability of motor and sensory brain regions to SSD (Kochunov, Ganjgahi, et al., 2016; Weinberger, 1996; Weinberger & Lipska, 1995). We hypothesize that by considering findings across the whole brain, RVI accentuates the regional effects specific to SSD. Therefore, higher RVI values are identified in the individuals with more severe patterns of neurodevelopmental damage, who, in turn, are more vulnerable to developing cognitive deficits and negative symptoms.

In Kochunov et al. (2020), presented in this issue, we show that RVI can be calculated as a multimodal index by considering cortical thickness, subcortical gray matter volumes, and white matter microstructure measurements. Combining phenotypes across diverse neuroimaging modalities to derive a meaningful index of vulnerability is challenging, but the ENIGMA‐schizophrenia findings provided a common denominator to combine these data. We first showed that RVI derived from cortical gray matter thickness, subcortical gray matter volume, and white matter integrity can inform patient–control differences and provide insight into the timeline for establishing these deficits in SSD. Elevated cortical RVI was readily detectable in the early diagnosis group (≤5 years since diagnosis) and remained stable with illness duration. This suggests that cortical deficits may develop before the onset of illness and do not change with illness duration. In contrast, white matter RVI was significantly elevated between early and chronic patients, suggesting ongoing illness progression. However, the multimodal RVI showed both the highest effect sizes among all measurements for all groups and was higher in chronic patients. While these findings are preliminary and are based on cross‐sectional analyses, they demonstrate the potential for translating ENIGMA patterns to the individual level. We expect that novel analytic approaches, including machine learning, will take advantage of the ENIGMA datasets to derive more comprehensive measures that translate statistics from a large group to make predictions about an individual.

### 4.2 ENIGMA‐DTI: Facilitating cross‐diagnostic analyses

The patterns of patient–control deficits derived using the ENIGMA‐DTI workflow by neuropsychiatric disorder‐oriented workgroups provide a “bottom‐up” approach to evaluate the “integrative” versus “diagnostic silos” heuristics in neuropsychiatric research (Bzdok & Meyer‐Lindenberg, 2017; McEwen, 2017). The integrative heuristic argues that risk factors, including genetics, stress, and others, are shared across major neuropsychiatric illnesses, while the diagnostic silos heuristic argues for separation of etiopathological factors while accepting potential co‐morbidity of these illnesses. Big data psychiatric genetics research provides evidence for the integrative nature of mental illness by showing strong genetic correlation (*ρ*_G_ = 0.5–0.7) among the risk loci for a range of common neuropsychiatric disorders and a significant overlap in PRS across SSD, BD, MDD, and OCD (Brainstorm et al., 2018; Cross‐Disorder Group of the Psychiatric Genomics, 2013; Docherty, Moscati, & Fanous, 2016). The combined efforts of the ENIGMA working groups provide us with the opportunity to examine the overlap in deficit patterns across disorders and to compare them to deficit patterns in chiefly genetic disorders (22q11 deletion syndrome) and/or with chiefly acquired (TBI) conditions.

**FIGURE 2:**
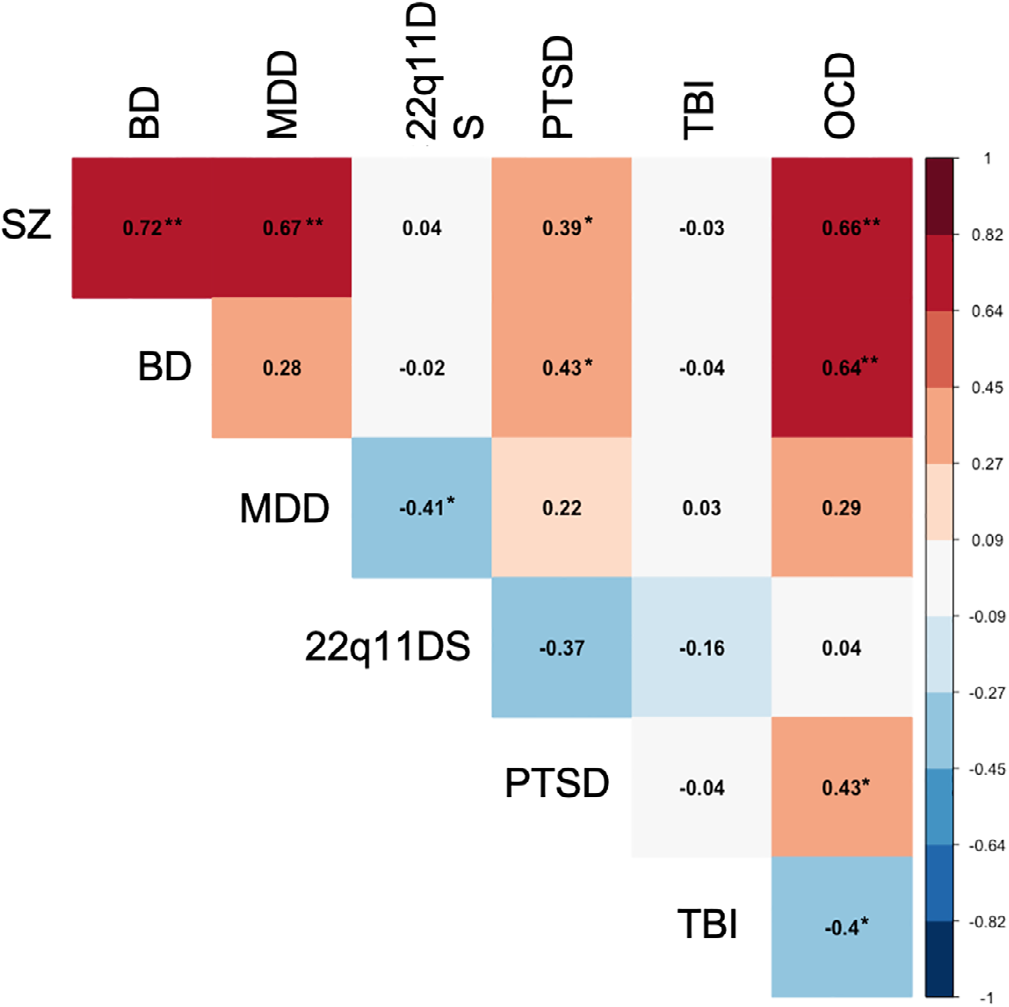
The correlation in regional deficit patterns among common neuropsychiatric disorders. **Indicates strong correlation coefficients. *Indicates moderate correlation coefficients

**FIGURE 3:**
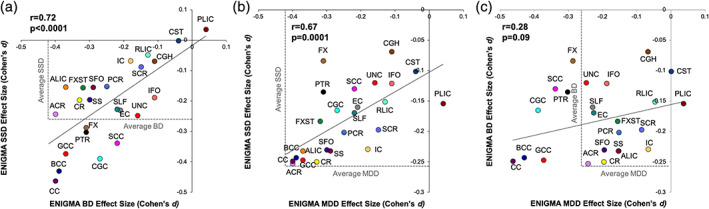
The scatter plot of regional effect sizes for (a) BD versus SSD; (b) MDD versus SSD, and (c) MDD versus BD. BD, bipolar disorder; MDD, major depressive disorder; SSD, schizophrenia spectrum disorder

The patterns of the effect sizes of the patient‐control differences showed strong correlations in regional effects sizes between SSD and BD (*r* = 0.72), SSD and MDD (*r* = 0.68), and SSD and OCD (*r* = 0.66) (Figure 2). The regional effect sizes were also strongly correlated between BD and OCD (*r* = 0.64) but not between BD and MDD (*r* = 0.28); nor MDD and OCD (*r* = 0.29) (Figures 2 and 3 **)**. The SSD, BD, and MDD had a striking similarity in the negative effects these illnesses have on the association and commissural tracts: both anterior (ACR, BCC, and GCC) and posterior (*sagittal stratum* [SS] and *posterior corona radiata* [PCR]) tracts. A notable difference was the integrity of the FX and FX/ST tracts that showed significant deficits in SSD and BD but not in MDD (Figure 3, Table 2). This suggests some anatomical specificity and partially replicates the findings of shared genetic risk factors among SSD, BD, and MDD (Brainstorm et al., 2018; Docherty et al., 2016). Strong correlations in regional effect sizes between SSD and BD (*r* = 0.75) and SSD and MDD (*r* = 0.82) were later replicated in COCORO data, however, the MDD and BD patients also showed a strong correlation in that cohort (*r* = 0.73) (Koshiyama et al., 2019). The deficit pattern of PTSD showed moderate correlation with the deficit pattern of BD (*r* = 0.43), OCD (*r* = 0.43), and SSD (*r* = 0.39) and a very weak correlation with MDD (*r* = 0.22). This further supports anatomical specificity of the white matter deficits and partially replicates genetic correlation patterns among these illnesses (Brainstorm et al., 2018). We observed no significant correlation between disorders with a strong genetic component (SSD, BD, MDD, PTSD, OCD, and 22q11) and TBI—which is presumed to have mainly causes of acquired injury and environment, though individual genetics likely affects the recovery and preexisting psychiatric disorders are associated with a worse outcome after TBI (Gerring et al., 1998). However, we observed a moderate negative correlation between TBI and OCD (*r* = 0.40) but this finding is difficult to interpret.

**FIGURE 4:**
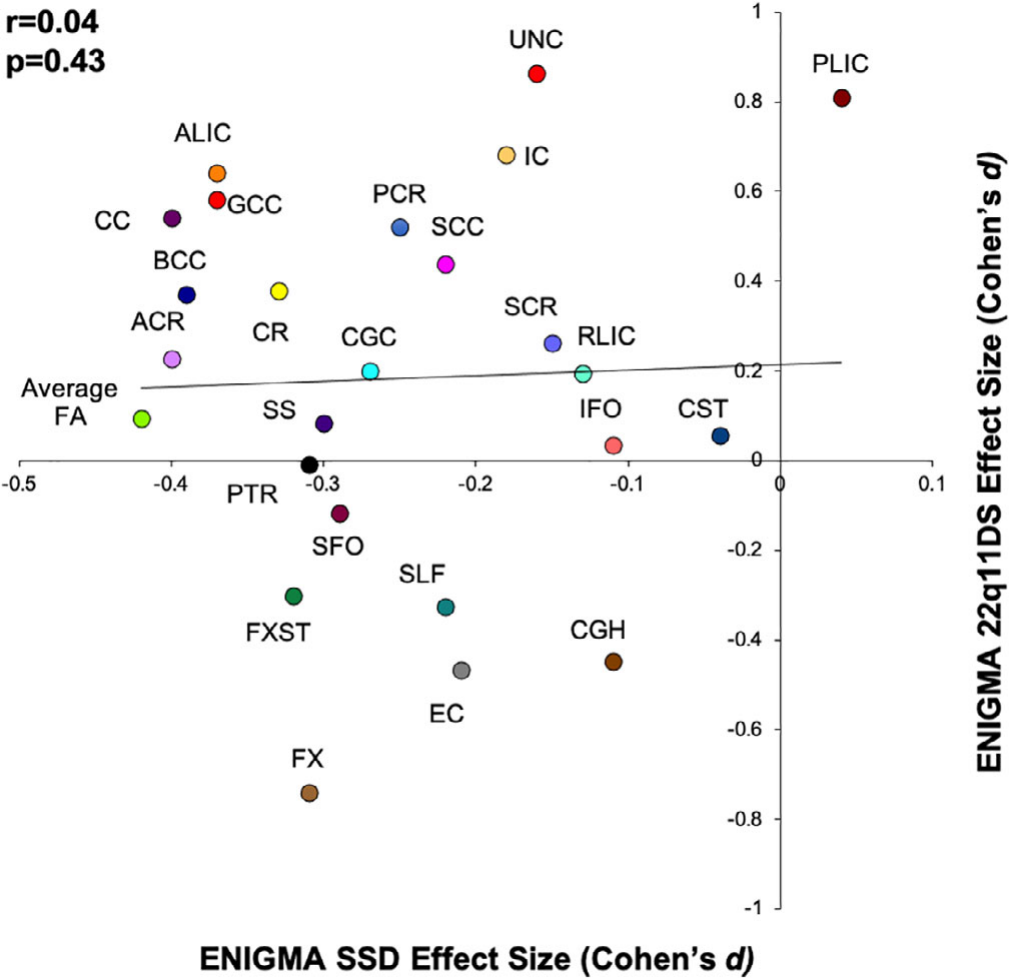
The scatter plot of regional effect sizes for 22q11DS versus SSD. Notable is high negative effect size in the Fornix (FX) 22q11DS that overlaps with negative effects of this tract in SSD. SSD, schizophrenia spectrum disorder

We observed no correlation (*r* = 0.04) between the 22q11 deletion pattern of regional effect sizes and that of SSD (Figure 4). 22q11 deletion is used as a developmental and genetic animal model for SSD (Mancini et al., 2019; Sumitomo et al., 2018) because people born with this deletion are 20–30 times more likely to develop psychosis. In striking similarity with SSD, the onset of psychosis is preceded by development of cognitive deficits, chiefly in the verbal learning and working memory domains (Vorstman et al., 2015). While subjects with the 22q11 deletion showed higher FA values in frontal areas, both SSD and 22q11 showed significantly lower integrity of the FX and FX/ST tracts (Figure 4), which is supported by findings of lower hippocampal volumes in both conditions compared to controls. This difference in regional deficits is also mirrored by the pattern of cognitive deficits between the two conditions. The deficits in processing speed are pervasive in SSD and are linked to lower integrity of associative white matter tracts (Kochunov et al., 2010; Kochunov et al., 2017), but these deficits are minored in 22q11 deletion syndrome. Conversely, both disorders showed significant deficits in verbal learning and working memory domains (Chawner et al., 2017; Vorstman et al., 2015).

**FIGURE 5:**
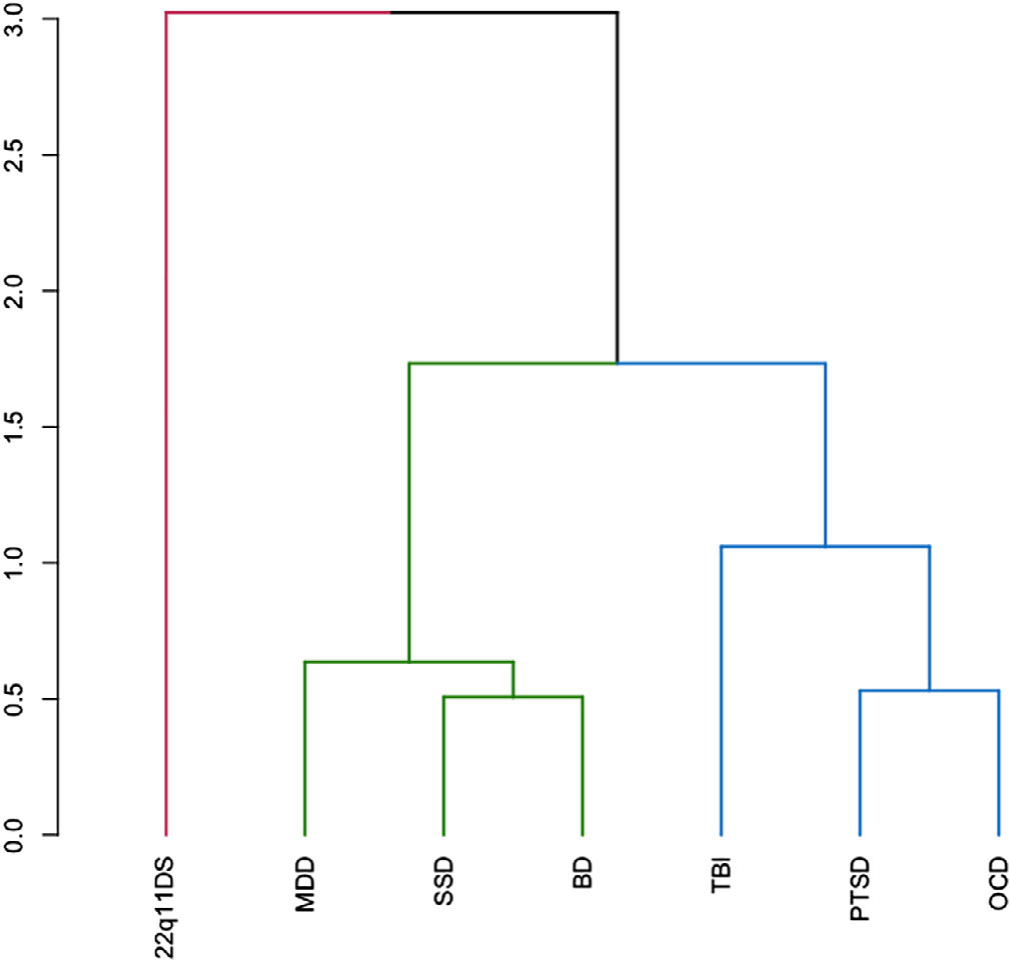
Hierarchical clustering of white matter deficit patterns across neuropsychiatric illnesses ascertained by ENIGMA. ENIGMA, Enhancing Neuro Imaging Genetics through Meta‐Analysis

To summarize the regional deficit data, we performed a hierarchical clustering analysis and measured the Euclidean distance among clusters (Figure 5, Table 3). Ward's minimum variance method was used to cluster the illness‐specific patterns based on the half‐square Euclidean distance among the deficit vectors. The disorder patterns were separated into three clusters based on their proximity. MDD, SSD, and BD were clustered together with the average distance between them of 0.56 ± 0.07. PTSD and OCD likewise were clustered together with TBI with an average distance between them equal to 0.73 ± 0.28. The pattern for 22q11 deletion syndrome was given its own cluster based on large distances from the MDD, SSD, and BD (distance = 2.9 ± 0.09) and PTSD, OCT, and TBI (distance = 2.36 ± 0.14).

**TABLE 3:** Euclidean distance among illness‐specific patterns of white matter deficits identified by the hierarchical clustering analysis

	SSD	BD	MDD	22q11DS	PTSD	TBI
BP	0.5063376					
MDD	0.5643102	0.6361531				
X22q	3.0234198	2.9166163	2.8504258			
PTSD	1.2727376	1.1126883	0.8718744	2.355787		
TBI	1.7329883	1.5730065	1.333859	2.2347175	0.6215351	
OCD	0.8618856	0.7279523	0.567146	2.513722	0.5301395	1.059763

Abbreviations: BD, bipolar disorder; ENIGMA, Enhancing Neuro Imaging Genetics through Meta‐Analysis; MDD, major depressive disorder; OCD, obsessive–compulsive disorder; SSD, schizophrenia spectrum disorder; PTSD, posttraumatic stress disorder; TBI, traumatic brain injury.

### 4.3 Limitations

This summary of ENIGMA cross‐disorder analyses demonstrates significant limitations of the biological interpretations that can be derived from DTI data within and across disorders. We observed that patient‐control differences can be both negative and positive indicating that neuropsychiatric conditions are associated with both lower and higher FA values in affected individuals. This signifies the general limitation of the DTI approach to quantify diffusion behavior of water (Basser & Pierpaoli, 1996). FA is a convenient statistical parameter produced by fitting a tensor that assumes a nonanisotropic Gaussian diffusion process and does not carry explicit biological information. While FA is often interpreted as an index sensitive to the degree of axonal myelination (Song et al., 2003; Song et al., 2005), it is neither a direct nor a specific measurement (Beaulieu, 2002). There are physical limitations to the assumptions of a multivariate Gaussian model used by DTI to approximate the diffusion in complex biological tissues. Chief among them is that this assumption is only successful at modest diffusion weighting (b‐values up to ~1000 s/mm^2^). At higher diffusion weighting, the diffusion decay cannot be approximated by a monoexponential fit that suggests a complex, multicompartmental nature of this signal (Assaf & Cohen, 1998; Clark, Hedehus, & Moseley, 2002; Kochunov, Chiappelli, & Hong, 2013; Kochunov, Chiappelli, et al., 2014; Wu, Field, Duncan, et al., 2011; Wu, Field, Whalen, & Alexander, 2011). These non‐Gaussian diffusion components (Novikov Dmitry, Kiselev Valerij, & Jespersen Sune, 2017; Novikov, Fieremans, Jensen, & Helpern, 2011) may both carry important information relevant to a disorder (Kochunov, Rowland, et al., 2016) as well as affect the fit of DTI model due to incomplete quantification of the diffusion process (Kochunov, Chiappelli, et al., 2014).

## 5 CONCLUSION

The ENIGMA‐DTI workflow was developed for imaging genetic analysis and validated by demonstrating uniform and reproducible heritability patterns across regional phenotypes. It was used across multiple brain disorders by ENIGMA workgroups and other studies for its ability to run the same analysis protocol worldwide, thus allowing multiple regional phenotypes to be aggregated and to deduce salient, consistent, and robust deficit patterns across illnesses. The regional deficits patterns published by ENIGMA in SSD and BD were already replicated in independent cohorts across the world, with the MDD pattern showing partial replication. ENIGMA deficit patterns can also be used to measure the agreement between an individual's brain scans and the aggregated patterns for each illness, offering a similarity metric to the canonical signatures observed in each disorder. Data across neuroimaging modalities can be combined into a multimodal index of individual vulnerability to various disorders. Such metrics may represent potential biomarkers for pharmacological studies of agents that aim to shift an individual away from the established pattern that is characteristic of a given disease. ENIGMA is equipped to run standardized analysis pipelines across disorders. Therefore, the similarity in deficit patterns across the major neuropsychiatric conditions can be readily assessed. The overlap and uniqueness in disorder‐specific white matter deficit patterns were consistent with the genetic correlation of risk loci for common neuropsychiatric disorders.

## CONFLICT OF INTEREST

P.M.T. and N.J. are MPIs of a research related grant from Biogen, Inc., for research unrelated to the contents of this manuscript. C.R.K.C. has received partial research support from Biogen, Inc. (Boston, USA) for work unrelated to the topic of this manuscript. O.A.A. is a consultant to HealthLytix, Speakers honorarium from Lundbeck. In the past 3 years, D.J.S. has received research grants and/or consultancy honoraria from Lundbeck and Sun. L.E.H. has received or plans to receive research funding or consulting fees on research projects from Mitsubishi, Your Energy Systems LLC, Neuralstem, Taisho, Heptares, Pfizer, Sound Pharma, Takeda, and Regeneron. All other authors have no conflict of interest to declare.

## Data Availability

Data Sharing: The data reviewed by this editorial are available from the manuscripts that published it.
